# From the Point of View of the Chickens: What Difference Does a Window Make?

**DOI:** 10.3390/ani11123397

**Published:** 2021-11-28

**Authors:** Elaine Cristina de Oliveira Sans, Frank André Maurice Tuyttens, Cesar Augusto Taconeli, Ana Silvia Pedrazzani, Marcos Martinez Vale, Carla Forte Maiolino Molento

**Affiliations:** 1 Animal Welfare Laboratory, Federal University of Paraná, Rua dos Funcionários, 1540, Curitiba 80035-050, Paraná, Brazil; anasilviap@yahoo.com.br (A.S.P.); carlamolento@ufpr.br (C.F.M.M.); 2 Flanders Research Institute for Agriculture, Fisheries and Food (ILVO), Scheldeweg 68, 9090 Melle, Belgium; frank.tuyttens@ilvo.vlaanderen.be; 3 Faculty of Veterinary Medicine, Ghent University, Salisburylaan 133, 9820 Merelbeke, Belgium; 4 Department of Statistics, Federal University of Paraná, Rua Evaristo F. Ferreira da Costa, 408, Curitiba 81531-990, Paraná, Brazil; cetaconeli@gmail.com; 5 Department of Animal Science, Federal University of Paraná, Rua dos Funcionários, 1540, Curitiba 80035-050, Paraná, Brazil; marcos.vale@ufpr.br

**Keywords:** artificial light, behaviour, dark side, environment, glass window, natural light, poultry, preference test

## Abstract

**Simple Summary:**

Light is an important environmental factor in many aspects for broiler chickens, such as behaviour and physiology, and welfare may be compromised when they are reared under low illuminance. We aimed to investigate what broiler chickens prefer when given free choice between a barn side with artificial lighting only as opposed to the other barn side with natural light through glass windows and artificial light. Environmental indicators and external conditions were monitored inside and outside the experimental barn, as well as chickens’ preference regarding location in each side of the barn and their behavioural repertoire. Chickens preferred the barn side with natural and artificial light from 18 days onwards, after the heating light was removed. Chickens’ behavioural repertoire changed according to barn side and their ages, expressing more natural behaviours and activity in the barn side with natural light. In summary, the birds indicated that natural light from windows makes a relevant difference in their lives, as it is what they choose when the only other option is the same in-barn environment with only artificial lighting.

**Abstract:**

We aimed to investigate what broiler chickens prefer when given free choice between a barn side with artificial lighting only as opposed to the other barn side with natural light through glass windows and artificial light. Eighty-five 1 day-old male Cobb 500 broiler chickens were divided into 10 pens; half of each pen area was provided with only artificial light (OAL) and the other half with natural and artificial light (NAL), and birds were free to move across sides. Environmental indicators and external conditions such as temperature, relative humidity, air velocity, ammonia and illuminance were monitored inside and outside the barn. Chickens’ preference was registered each three days, divided in categories: I (at 9, 12, and 15 days), II (at 18, 21, 24, and 27 days), and III (at 30, 33 and 36 days). The effect of the interaction between environmental indicators and week was statistically different only for illuminance. Chickens preferred NAL to OAL from 18 days onwards (II *p* < 0.001; III *p* = 0.016). Drinking (*p* = 0.034) and exploration or locomotion (*p* = 0.042) behaviours were more frequent, and “not visible” behaviours (*p* < 0.001) were less frequent, in NAL. Foraging was the only behaviour with an interaction effect between age category and light treatment, as birds during period II expressed this behaviour more frequently in NAL than OAL (*p* = 0.003). For our experimental conditions, the chickens preferred NAL from 18 days of age onwards, when the confounding effect of the heating light was removed, and their behavioural repertoire was also different according to each side of the barn and to their ages.

## 1. Introduction

In general, broiler chickens are intensively reared worldwide in large flocks confined in indoor houses where food, water and environmental control are available to provide for their basic physiological needs [1]. However, considering bird evolutionary history, conditions provided by the production chain are far apart from that found by chickens in a natural life. In nature, they are exposed to a variety of circumstances and environmental conditions which include the day length and photoperiod [1,2].

Broiler chickens subjected to commercial management are typically housed in dim lighting because it is presumed to improve productivity and feed conversion efficiency, reducing overall activity and injurious pecking [3,4]. Such inactivity caused by low illuminance is likely related to an apathetic state, as responsiveness to many stimuli seems reduced, even though it is commonly confounded with a calm state [5]. In fact, light is an important environmental factor for the animals [4,6]. More specifically for broiler chickens, lighting quality and intensity affect their behaviour and physiology [3,6,7,8,9,10]. Natural lighting as a positive factor for bird welfare is a common assumption. However, it is not clear whether this assumption holds when natural light is offered through glass windows and, thus, in a different constitution as compared to outdoor natural lighting. It remains true, though, that natural lighting through windows may provide a dynamic range of illuminance levels in different areas within the house, with considerably higher intensities as compared to the regular artificial lighting recommended for birds. Thus, the potential for enrichment of the perceived environment and, consequently, for improving bird welfare through barn windows [11] seems to warrant further investigation. The birds do express more natural behaviour and are more active compared to birds not exposed to natural light [11]. Although there are types of lamps that can offer the same characteristics as natural illuminance, such as bulbs supplemented with ultraviolet (UV) light fixtures [12], these technologies are not widely used in Brazilian chicken barns, for which a variety of lamp types is observed, such as incandescent and fluorescent lamps [13,14]. In this case, according to the light source type, artificial illuminance may differ from natural light in terms of light colour, intensity, photoperiod, and flicker [9], and these characteristics may influence bird preferences [15]. Moreover, worldwide recommendations for illuminance inside the barns accept extremely low levels of 20 lux (lx) [16,17] and this seems to represent an important subject to be discussed regarding broiler chicken welfare.

Vision is probably the dominant sense in domestic poultry, and the evolution of vision was determined, in part, by the natural light available [18]. The photoreceptive pigments in the retina allow birds to perceive colours in a more detailed way than humans [19]. Birds also have the ability to perceive ultraviolet (UV) light, with the spectral sensitivity below 350 nm [12,19], and may experience a better quality of vision in brighter environments [20]. In a natural scenario, UV light is important for birds in relation to orientation, foraging, calibration of their circadian clock, and sexual selection [21]. In intensive systems, according to glass types, the full passage of UV light is blocked, but windows may be an alternative for providing some UV wavelengths to chickens [11,22,23].

If birds perceive natural and artificial light in different ways, this may influence their behaviour. Manser [7] suggested that light intensities between 5 and 22 lx, currently used for broiler chickens and turkeys, may contribute to the decrease of their engaging in exploratory behaviour and social interaction, high prevalence of leg abnormalities, mortality, eye abnormalities, breast blisters in growing birds, and fearfulness. Surely, the study of behaviour is an important tool for the identification of relevant environments and devices to the animals, justifying the provision of adequate resources to the animals [24]. Preference tests suggest that most broiler chickens make consistent and rational choices associated with the environments that are associated with lower fear and stress responses [25,26]. However, there is a lack of studies about lighting preferences by the birds, and this is especially relevant nowadays, when there is an increase in the number of closed-houses [27]. There is an increasing number of companies replacing natural by artificial lighting, in systems that apply the minimal illuminance recommended for broiler chickens houses (20 lx) [16], or even less than the recommended minimum.

Although there are no public data regarding the proportion of each type of poultry house type in Brazil, broiler chickens in intensive systems are mostly raised in two main barn types [13,14]. The conventional system employs open-sided poultry houses, where the natural daylight may enter without passing through glass when their movable curtains are open; they are called conventional because they used to predominate in the Brazilian poultry meat industry. Lately, the closed-poultry house type is rapidly becoming more popular in Brazil, and it uses only artificial light. Open- and closed-sided poultry houses have positive and negative welfare aspects, which may also vary according to season [13,14]. However, the quantity and quality of the light available to the birds may be considered a major factor that differentiates these two barn types in terms of their animal welfare potential.

Our objective was to investigate the importance of the existence of windows in the barns by studying what the chickens prefer when given free choice between an area with only artificial lighting (OAL) and an area with natural and artificial lighting (NAL). Our hypothesis was that the NAL has a significant effect on animal behaviour and that it would be preferred by birds.

## 2. Materials and Methods

**Figure 1:**
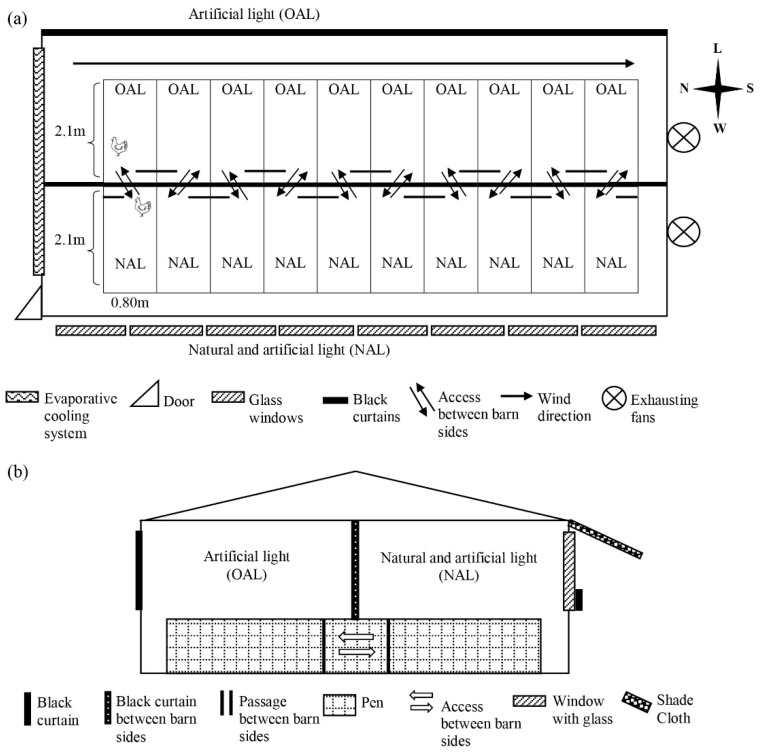
Experimental design of preference test seen from above (**a**) and from the barn entry side (**b**). The house was divided in two sides, one with Only Artificial Light (OAL) provide by LED lamps, and the other side, with Natural Light provided by glass windows and artificial light provided by the same lamp type and quantity (NAL), from January to February 2021, in the State of Paraná, South of Brazil.

**Figure 2:**
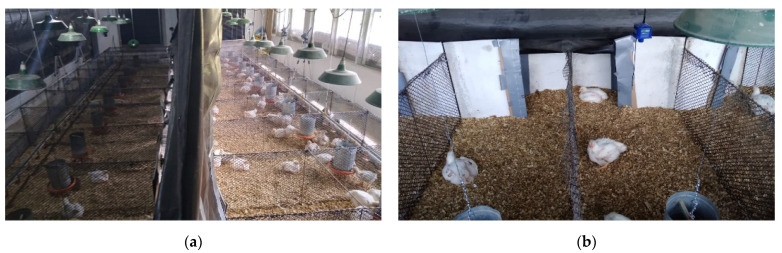
Overview of the inside of the house (**a**) both sides shown, Only Artificial Light (OAL) on the left, and Natural and Artificial Light (NAL) on the right; and (**b**) separation between sides constructed of black curtain and wooden panel, in a preference test performed from January to February 2021, in the State of Paraná, South of Brazil.

The study was conducted between January and February 2021, in an experimental broiler house measuring 10 m × 6 m × 2.5 m (Figure 1), of the Federal University of Paraná farm, Pinhais, Brazil (25°23′36.2″ S, 49°08′2.9″ W) at an altitude of 935 m. The house was built in a North-South orientation with 10 pens, each one with a total area of 3.36 m^2^ (0.80 m × 2.10 m). Eighty-five one-day-old male Cobb 500 broiler chickens were randomly distributed into ten pens, as groups of eight birds in five pens and of nine birds in the other five pens. The experimental design was planned for eight birds per pen and the additional birds were included to cover for eventual mortality throughout the experimental period. The experimental barn was longitudinally divided into a barn side with no windows and only artificial light (OAL), and the other side was built with a window throughout its lateral wall and received both natural and artificial light (NAL); pens were built transversally, so that half of each pen was in the OAL and the other half in NAL side (Figure 2a), resulting in 1.68 m^2^ per pen in each barn side. Ten LED lights were evenly spread across the entire pen areas, in both the OAL and NAL sides. The passage between NAL and OAL barn sides was always open, and the birds were allowed to move freely across the sides as they chose.

Artificial light was provided by Light Emitting Diodes (LED) white lamps of 9 W, 6500 K (correlated colour temperature), dimmable, with no UV or infrared emission, distributed along each side of the barn, suspended from the ceiling at a height of 1.50 m from the floor. In NAL sides, in addition to the same quantity and quality of artificial light as in OAL side, natural daylight was provided through eight windows along the west lateral wall of the barn, measuring 1.25 m × 0.95 m each, equipped with 8 mm colorless tempered glass. The use of glass is opposed to the more common open-sided barns in Brazil. According to the glass type, some UV wavelength may be blocked [22,23]; however, the glass was a necessary resource to maintain the control on internal environmental conditions other than lighting between barn sides, to ensure that bird preference was based exclusively on illuminance, without any interference of other factors such as temperature or relative humidity. Approximately ¾ of the window areas on the wall of the NAL side were shut by black curtains between 06:00 PM and 07:00 AM, and for the OAL side, the windows were totally closed by black curtains throughout the experimental period.

A black curtain was used in the center of the barn to separate the OAL and NAL sides (Figure 2a), installed from the ceiling down to 60 cm from the floor. Wooden separators filled this 60 cm close to the floor, and this wooden separation contained passages of 0.50 cm, which allowed for the birds to have free access to both sides of the pen (Figure 2b).

All the pens were equipped with the same quantity and quality of feed, litter, heaters, manual feeders and drinkers, and, from 10 d old onwards, nipple with cups drinkers. Infrared lamps of 240 V and 175 W, for both barn sides and all pens, were used to heat the birds. The heating lamps added, on average, up to 25 lx more in each pen. The pens were made by plastic mesh fence, to facilitate the passage of air. Two exhaust fans, one for each side of the barn, and one evaporative cooling system ensured appropriate temperatures in the entire poultry house. A polyethylene shade cloth was installed on the West side to decrease the direct solar incidence through the glass windows that was observed after 03:00 PM. Natural shadow was provided by trees on the East side of the house (OAL).

### 2.1. Environment Measurements

Environmental indicators were measured twice every day, at 10:00 a.m. and 03:00 p.m., for the duration of experiment. According to tests carried out before the start of the experiment, the environmental conditions were similar across the house. For this reason, we took measurements at two places indoors, in the middle of the house, on each barn side. The outdoor conditions were monitored for approximately 3 m in front of the barn main entrance, in a place with no coverage. The indoor environmental indicators were monitored at bird level, in the center of pen five in each barn side (OAL and NAL). Temperature, relative humidity, air velocity, and illuminance were measured using Lutron LM 8000A (Akso, São Leopoldo, Brazil). Ammonia concentration (NH_3_) was measured by SP2nd Portable Single-Gas Detector (Senko, Osan-si, Korea).

### 2.2. Experimental Design

On the first day of birds’ lives, five groups of birds were initially housed in the OAL side, and the other five groups in the NAL side. Birds had six days of adaptation, for learning between the barn sides offered within each pen, and avoiding any potential confounding effects due to fear of novelty or other factors related to the new environment initially faced by the animals. From day 7 on, each bird group was relocated every three days to the next pen located to their right, allowing for all the groups to stay for three days in each of the 10 pens available in the experiment. If a bird or a group of birds was in the OAL side, they were relocated to the next pen also in the OAL side; the same was done for the birds in the NAL side. Birds were only relocated after emptying the destination pen, thus avoiding contact between birds from different groups; birds in the last pen of the barn were relocated to the first pen, considering the barn door. This management allowed for testing whether there was a pen effect by separating it from group effects. The beginning of assessments only started after two days of the group change, allowing the birds to get used to their new pen. In case of mortality, birds were relocated as needed to maintain a minimum of eight birds per pen. Until 18 d, in both sides, the birds were exposed to 24 h of light and no dark periods, i.e., 24L:0D, on both side. After this period, the birds received a 16L:8D continuous lighting regimen. The switch was done in the following manner: after 14 d of age, heating lamps were turned on only during the night period; after 18 d of age, all heating lamps were removed and birds became exposed to complete darkness from 09:30 PM until 05:30 AM.

### 2.3. Bird Preference and Behaviour

We video-recorded both sides of two different pens per day, the number of birds in either OAL or NAL sides, and their behavioural repertoire, by fitting four video cameras, Canon Vixia HF R800 (Canon Inc., Zhuhai, China), one installed in front of each side of each two pens. Recordings started on day 9 and ended on day 36, always from 07:30 a.m. to 05:30 p.m., and were conducted every third day, totaling 10 d of observations with 100 h of video-recordings, with 20 pen observations. All the pens recorded were chosen at random, allowing for different pens and groups of birds to be recorded during the experimental period.

Birds’ preference was measured by the count of birds present in each side of the barn. Their behaviour was analyzed according to a predefined ethogram (Table 1), using the same video-recording. Both count of birds and behaviours were observed by scanning methodology, with instantaneous sampling every 1 h [28,29].

Bird health condition and mortality were checked daily. Birds with severe lameness that compromised their ability to drink and feed, i.e., scores 4 and 5 [30], were culled by cervical dislocation.

### 2.4. Statistical Analyses

Mortality and outdoor environmental conditions such as temperature, relative humidity, air velocity, NH_3_ concentration, and illuminance were analyzed by descriptive statistics. For the same environmental indicators, measured indoor and in both barn sides, linear regression models were fitted to test the main effects of house side (OAL or NAL) and age (from 1 to 6 weeks), in addition to the interaction effect. The Tukey’s test for multiple comparison was used to ensure a global significance level of *p* < 0.05, and the goodness of the fitted models was assessed through residual analysis using half-normal plots with simulated bands.

Bird preference and behaviour data were analysed by mixed regression models. Total counts of birds in OAL and NAL barn sides, which means the total of birds verified in each barn side, throughout the day for each pen, and the recorded counts, were considered as the response variable. The fixed effect of chicken age and the random effects of group of birds and pens were considered. Age was categorized according to period: I (at 9, 12 and 15 d old), II (at 18, 21, 24 and 27 d old), and III (at 30, 33 and 36 d old). A binomial generalized linear mixed model was initially fitted, but the residual diagnostics clearly indicated that it was inadequate. Then, to account for overdispersion verified in this experimental data, a beta-binomial mixed regression model [31] was adopted, which are useful for analyzing discrete rates, such as the proportions of birds verified in NAL and OAL barn sides throughout the day for each pen.

For each of the remaining behavioural variables, a beta-binomial mixed regression model was also fitted. In such cases, the fixed effects of age categories, side of the barn (OAL or NAL), and the corresponding interaction effect were evaluated. The variables group of birds (birds that were reared together during all experimental period), pen (10 boxes distributed throughout the barn sides), and pen/day (the exact group of birds in each pen for a specific day of behavioural observation) were considered as random effects; this last random effect was needed as the design included the rotation of bird groups across pens, thus allowing for the study of any pen effect without the confounding effects of bird group. The fitted models were successively simplified by removing the non-significant fixed effects, starting with the interaction effect, then the main effects of age class and barn side, and considered birds rates recorded in OAL and NAL barn sides, taking into account only the total number of live birds.

The model results were summarized through the estimated probabilities and corresponding confidence intervals (CI; 95%). The estimates and standard errors for the variance components of random effects are also presented. The age categories, when statistically significant, were compared using a multiple comparison procedure with properly adjusted *p*-values.

Statistical analyses were performed using the R software 4.0.2 [32] and conclusions were based on a significance level of *p* < 0.05. The contrasts of means for environmental indicators were estimated using the emmeans package [33]. The hnp package [34] was used for the residual analysis, and the plots were produced through the ggplot2 package [35]. The PROreg package [36] was used to fit beta-binomial mixed regression models for preference and behaviour analysis.

## 3. Results

From 2 d, it was observed that some chickens started to move spontaneously between OAL and NAL barn sides, and from 4 d old, at least one bird in each pen had already accessed both sides of the barn. Soon afterwards, from day 6, the number of birds crossing between barn sides became high. Thus, it was not necessary to intercede or teach the birds how to move between the barn sides.

The total mortality was 9.4% (8 of 85 birds). The main cause of death, for four of the eight birds, was associated with culling due to severe lameness. Other mortality causes indicated one bird with ascites and another bird with avian infectious bronchitis; the other two birds did not have their deaths investigated, and died at 7 and 13 d. Two birds, one per pen, were relocated to maintain eight birds per pen, and this procedure occurred before the birds were 15 d old.

### 3.1. Environmental Measurements

**Figure 3:**
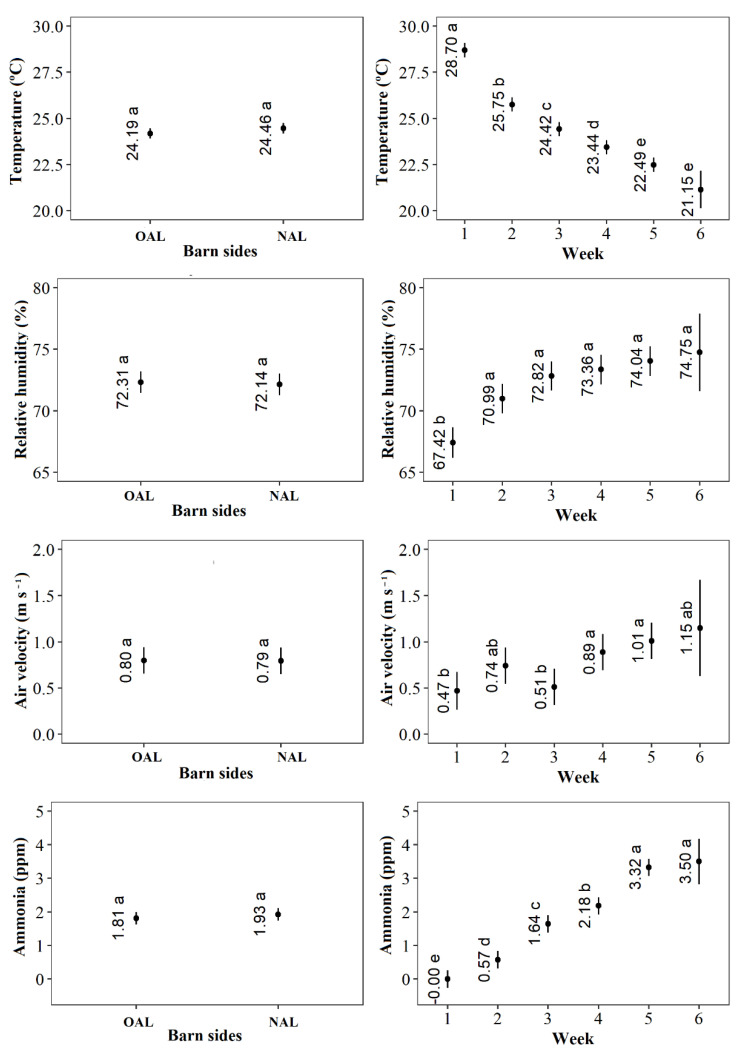
Estimated means and confidence intervals for indoor temperature (°C), relative humidity (%), air velocity (m s^−1^), and ammonia concentration (ppm), for Only Artificial Light (OAL) vs Natural and Artificial Light (NAL; left column of panels), and across weeks (right column panels). Data were collected twice daily from week 1 to 6, at 10:00 a.m. and 03:00 p.m., in a preference test performed from January to February 2021, in the State of Paraná, South of Brazil; means followed by the same letters do not differ (Tukey test, *p* < 0.05).

The average (min to max) values for outdoor environmental conditions during data collection periods were: temperature 25.5 °C (17.0 to 31.5 °C), relative humidity 72.6% (51.0 to 99.9%), air velocity 0.7 m s^−1^ (0.0 to 3.6 m s^−1^), illuminance 11,716 lx (2500 to >20,000 lx), and NH_3_ concentration 1.0 ppm (0.0 to 2.0 ppm). Results for indoor environmental measures showed minimal difference, and did not differ statistically between the OAL and NAL barn sides for temperature, relative humidity, air velocity, and NH3 concentration; however, overall differences across experimental weeks were observed (Figure 3).

**Figure 4:**
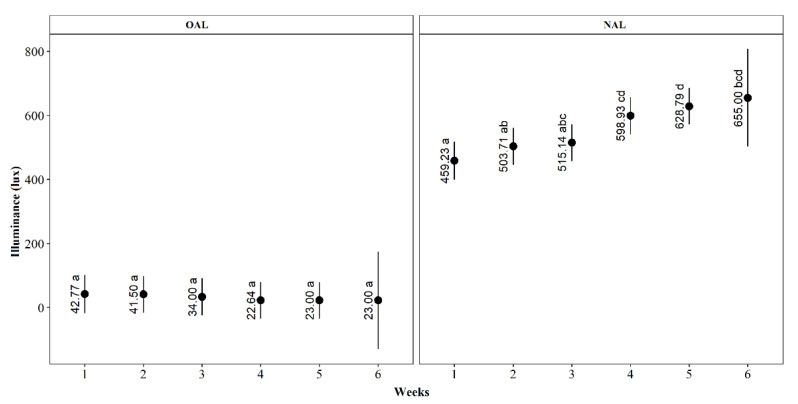
Means and confidence intervals for illuminance (lux) according to barn sides (OAL and NAL) across age from 1 to 6 weeks, in a preference test performed from January to February 2021, in the state of Paraná, South of Brazil; averages followed by equal letters do not differ statistically (Tukey test, *p* < 0.05).

Illuminance was the only indoor environmental indicator with a significant effect of the interaction between barn sides and weeks. Even though overall illuminance was significantly higher in the NAL side, it is clear that it significantly increased as weeks went by in the NAL side, while it remained constant throughout the period of six weeks for the OAL side (Figure 4). This increase in illuminance occurred due to a continuous period of rain, especially in the first three weeks of our experimental period. The average (min to max) values for illuminance during all weeks were 32.4 lx (22 to 44 lx) in OAL and 545.5 lx (280 to 900 lx) in NAL.

### 3.2. Bird Preference and Behaviour

**Figure 5:**
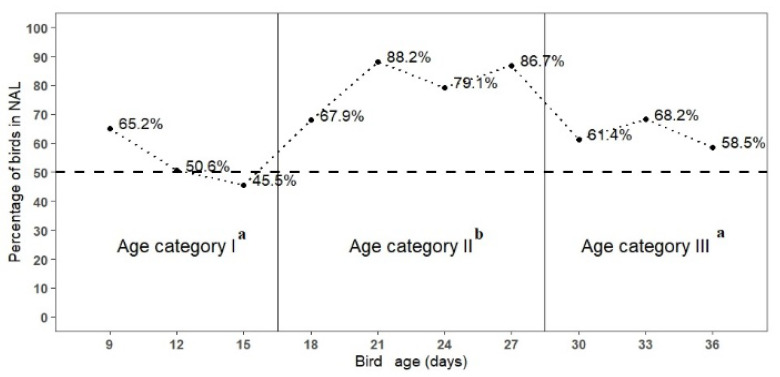
Percentage of broiler chickens observed in Natural and Artificial Light (NAL) barn side, according to bird age category (I at 9, 12, 15 d old; II at 18, 21, 24, 27 d old; III at 30, 33, 36 d old), in a preference test performed from January to February 2021, in the State of Paraná, South of Brazil; age categories followed by the same letters do not differ (*p* < 0.05); and dashed line indicates weekly values within age categories.

After the heating light was removed, from 18 d of age onwards, results showed in Figure 5 suggest that broiler chickens preferred NAL to OAL. This preference was significant for age categories II and III (Table 2). Results regarding birds’ preference by age categories, not included in the tables, show that birds in period II expressed higher preference for NAL when compared with period III (*p* = 0.007). Averaged for all ages, 32.9% of the birds were seen in OAL and 67.1% in NAL.

Results regarding feeding and comfort behaviours showed no window effect (Table 3), but a significant effect of the age categories. The difference in frequency of feeding behaviour was significant between period I vs. period III (*p* = 0.020). The frequencies for comfort behaviour were different across all the three age categories: period I vs. period II (*p* = 0.002), period I vs. period III (*p* < 0.001), and period III vs. period II (*p* = 0.036). The presence of the window was a significant factor for drinking (*p* = 0.034) and exploration or locomotion behaviours (*p* = 0.042), which were more frequent in NAL. The category “not visible” showed higher counts in OAL (*p* < 0.001), and the only behaviour observed was “any behavior that was not identified, due to birds standing in the shielded part of passage ways between barn sides due to unsatisfactory recording angle”. There was no significant effect for inactive behaviour (*p* > 0.05) and this was the most common behaviour in both OAL (47.0%) and NAL (44.6%) barn sides.

There was a significant effect for the interaction between windows and age categories for foraging behaviour (Table 4): when chickens were younger, in period I, they foraged more frequently in NAL than OAL (*p* = 0.003), while for the other two age categories, there was no difference. Considering the behaviour observed when the chickens were on the NAL side, birds in period I foraged more frequently than when they were in age category II (*p* < 0.001); the difference remained significant when birds in period I were compared with the same birds in period III (*p* = 0.009). There were no differences across the age categories when the chickens were observed in the OAL barn side.

## 4. Discussion

In general, our results showed that, after the heating light was removed, from 18 d of age onwards, broiler chickens preferred NAL to OAL. This preference was significant for age categories II and III. The chickens spent more time drinking, exploring and moving, and foraging in NAL than OAL. Inactive (the most commonly observed behaviour), feeding, and comfort behaviour did not differ significantly between OAL vs. NAL, only according to bird age category.

Regarding birds’ preference, our results are in agreement with other studies which showed that birds chose environments with higher illuminance and also expressed other changes in their behavioural repertoire due to differences in light intensity [37,38,39,40], and in our study we observed average of 32.4 lx in OAL and 545.5 lx in NAL. According to Lima and Silva [41], the absence of natural light, especially in closed-sided houses, may limit the expression of natural behaviours, with negative impacts on chicken welfare. Prescott et al. [8] strongly recommend a combination of natural daylight and artificial light for poultry barns. These considerations regarding the use of natural light are dependent on the importance of this choice for the birds themselves, with a potential to improve their welfare which tends to be proportional to the importance of natural light from the point of view of the birds. Our results especially contribute to the understanding of the birds preference, as the only internal environmental indicator that showed significant difference between OAL and NAL barn sides was illuminance. This represents an overall response of the birds to light conditions which warrants further studies, to understand the importance of other light characteristics, such as wavelength or spectrum variances. The light intensity is one of the most studied light characteristics for broiler chickens [20,37,39,40], and the bird preference for higher illuminance encouraged behaviours such as drinking, exploration or locomotion, and foraging in our study. Regarding other internal environmental measures, our results for relative humidity were not ideal, especially from the third week onwards. Even though values were close to the acceptable range between 45–70% [16], this non-compliance may be a welfare problem for the animals. However, this situation most likely did not influence the choice of birds (NAL or OAL), because relative humidity was the same on both barn sides.

Solar radiation reaching the earth surface is divided into infrared radiation, visible light, and UV; the latter is divided into three types according to wavelength: UVA (315–400 nm), UVB (280–315 nm), and UVC (100–280 nm), but 99% of the UV that reaches earth is UVA [42]. The solar radiation types that effectively reach individuals vary according to existence and type of eventual physical barriers. Tempered glass of 4 mm may block up to 28.4% of UV light from reaching the individuals [22], and 8 mm, 54.5% [23]. The glass type may also block at least 90% of wavelengths under 350 nm ([22,42]. However, windows with glass allow both visible wavelengths and a small amount of UV to pass to inside the houses [11] and, thus, alter chicken behaviour [43]. In the NAL barn side, birds may have received UV light that was not available in the OAL side. This may have motivated their preference, as poultry have a fourth retinal cone photoreceptor that allows them to see in the UVA wavelength (315–400 nm) [19]. Birds exposed to some UV light may have decreased stress susceptibility and fear responses than those raised without UV [12], showing that the illuminance of poultry houses can be improved in several aspects.

Regarding lamp types, LED bulbs with colour temperatures over 5000 K, called cold [44], contain more blue than warm white light [45], and in our study 6500 K lamps were used in both NAL and OAL barn sides. Understanding light temperature is relevant to our study as, in addition to light intensity, birds may also choose specific colour temperature [9,18]. In our study, the OAL light source was similar in terms of colour temperature, as measured in degrees of Kelvin (K), to the average daylight that birds were searching for by moving to the NAL side of the barn, suggesting that the light intensity, as measured in Lux (lx), may have been the main driver for the preference.

New lighting technologies are currently being developed as potential replacements for incandescent light sources, and some sources may be better to the welfare of broiler chickens [46]. However, our results suggest that the exposure to natural lighting may be an ideal solution according to the preference of the birds. This warrants further preference studies with different types of artificial light bulbs, as well as asking the birds how strong their preference is, through motivation tests. Considering the higher visual perception capacities that birds have as compared to humans, it seems relevant to explore light characteristics in addition to intensity to better understand what the birds are responding to when they express their preferences. In future research, the real perception of birds in relation to illuminance may be further studied. Although the differences in perceived light intensity by birds, known as Clux or Gallilux, may be estimated by adding between 20–25% in relation to lx, i.e., 25 Clux = 17.4 lx [47,48,49], it is important to study light from a bird perspective with more precision technology.

Bird preferences may be influenced not only by barn sides and their characteristics regarding light, but by their natural behaviours [24]. A special consideration is that chickens are social animals, and bird preferences may be influenced not only by individual choices, but also by their social nature and its effects, such as social facilitation [8,29,50]. Because of social facilitation, the birds tend to behave as a social unit, where most members exhibit the same behaviour at the same point in time [5]. Thus, the higher number of chickens in NAL side may have acted as an additional force for more birds to migrate to this side.

Bateson and Seanurne-Way [51] suggested that when birds were exposed to constant light, the elicitation of social behaviour became more likely. Our results seem to reinforce the statement that a place with higher illuminance fosters group formation that may be positive for the animals. Recognition between individuals is also part of the social interaction process, and this characteristic may be affected when birds are reared in very low illuminance [3,7,18]. Although in our study we have not observed any aggressive behaviour among birds, according to Porter et al. [52], chicks that had been housed in pairs in the dark showed no evidence that they discriminated between familiar and unfamiliar test partners. Thus, the NAL side may also have provided a better recognition of individual birds and, consequently, this may be potentially considered an additional factor explaining bird choice. Collins et al. [53] reinforce the importance of vision in key behaviours such as feeding and social behaviour in poultry, and suggest that the birds may experience lower welfare as a result of their lack of sight. Therefore, when birds choose the NAL barn side, they may be making choices to favour their natural social interaction behaviours.

The birds spent a considerable proportion of their time in the OAL barn side, and this choice should also be considered. Although higher light intensity has been associated with increases in activity levels and improvement in leg health of commercial broiler chickens [11], birds should have access to different types of illuminance, so that they can choose according to their preferences. As it is recognized that enrichment strategies should be provided for chickens [16], illuminance may follow the same principle. An adequate density associated with the availability of different types of light environments may reduce bird crowding, preserving their safety and health. Thus, the illuminance distribution must be adequate, avoiding a contrast between the lightest and darkest points of more than 20% [54]. The provision of areas with reduced light intensity for resting and other activities has been suggested before [40]. On farms where windows are provided, resting behaviour occurs more often in areas with lower light intensities, whereas active behaviours occur in areas with higher light intensities, but it is up to the birds to choose [55]. This pattern of light intensity choices is expected for diurnal animal species. Vergneau-Grosset and Peron [56] recommended that when exposing an animal to UV light, it is important to provide a hiding place or shade; in our study, both the passage way between each barn side and the OAL side may have fulfilled this function. Other negative effects of excess UV radiation intensity may be observed in both natural and artificial light sources, and revolve around the occurrence of burns in animals and behavioral changes, such as increased stress or incidence of severe feather pecking [56,57]. During the experimental period, none of these characteristics were observed in our birds, which agrees with the probably low UV exposure through the glass window.

In our study, the birds showed preference for the NAL barn side only from 18 d onwards. The association of this preference with bird age was also observed during other preference test, when chicks spent most time in the brightest light (200 lx) at 2 weeks, and at 6 week the birds preferred the environment with dimmest light (6 lx) [37]. The age for birds to begin expressing light preferences coherently coincides with the total removal of the heating lamps. Although this type of lamp is not suitable for lighting, it was responsible for adding up to 25 lx in each pen, which may have acted as an important confounding effect for birds to detect the lighting differences between barn sides. In addition, according to Gunnarsson et al. [29], early exposure to natural or artificial light might have an effect on later preference for light type and on the behaviour of the birds, even after a house transition. Therefore, the birds may have grown habituated with the illuminance from the heating lamps and, after their removal, they may have been obliged to make new choices, as their early life light experience became absent. In addition, the heating lamps may have provided an early imprinted association between light intensity and heat, reinforcing a positive perception of light by the birds. Even though it was not possible to identify the exact reason for bird preference for the NAL barn side, most possible explanations seem coherent with the more natural characteristic of the lighting on this side of the barn. Our hypothesis is that the windows tend to be closer to meeting the birds’ basic needs in relation to light and, thus, tend to increase animal welfare. Examples of such needs include the establishment and maintenance of social hierarchies, social encounters, group aggregation and peer recognition.

Results regarding chicken behaviours showed that the frequencies varied according to barn sides (drinking, exploration and locomotion, and not visible), and bird age categories (feeding and comfort). The behaviours of drinking and exploration and locomotion showed higher frequencies in the NAL side, and the category of not visible birds was more frequent in the OAL side. Davis et al. [37] observed that broiler chicks performed more feeding, drinking, and locomotion behaviours in the brighter environments. However, for Deep et al. [58] light intensity had no effect on expression of drinking behaviour. Adding further evidence to this discussion, our results indicate that providing windows increases the behaviour repertoire, a fact observed in previous studies. Sans et al. [13,14] observed that broiler chickens reared in open-sided houses, with natural light provided by no-glass windows, but with curtains during summer/autumn, showed higher relative frequencies for exploration behaviour when compared with birds in closed-sided houses; during the winter, there was a higher frequency for drinking and a lower inactivity. Thus, our results suggest that, even with eventual changes in natural daylight characteristics due presence of glass in the windows, it remains possible to observe a potential improve in bird welfare as the increase of activities considered important for the birds, i.e., social activities in the NAL barn side. Furthermore, windowed industry barns in Brazil do not fit glass barriers, and this was an experimental resource to control for other in-barn environmental conditions such as temperature and relative humidity, in order to study the specific effect of lighting. These results reinforce that, when given the opportunity, birds prefer to perform their behaviours in an environment with natural daylight or, minimally, higher levels of illuminance than those provided inside the barns with only artificial lighting.

The exploratory or locomotion behaviours, observed in higher frequency in NAL side, tend to be viewed as positive behaviours, because may increase the birds’ activity and improve the interactions between the birds and their environment [1]. However, if the house is not stimulating, as birds age they may get bored and reduce exploratory behaviour [1]. It seems important that broiler chickens are reared in stimulating poultry houses, with adequate lighting characteristics that allow for the birds to perform activities which are essential for their welfare.

Foraging was the only behaviour for which a significant interaction effect between window and age categories was present, indicating that birds foraged more, when were younger, in the NAL than the OAL barn side. One of the reasons for the interaction with bird age may be the appearance of locomotor problems which tend to become more severe as birds age, in addition to increasing body weights and non-stimulating environments [16,51]. Alvino et al. [4] also observed that foraging was affected by light intensity, and broiler chickens in the 5 lx treatment spent significantly less time performing this behaviour than when the light intensity was 50 and 200 lx. Foraging, exploration, or locomotion are important behaviours, since they involve actions related to knowing the environment and searching for feed [16]. According to Manser [7], newly hatched birds, both domestic poultry and turkeys, may die of malnutrition if they have difficulty in seeing the feeders due low light intensity, which may reduce overall activity, reducing the chances of foraging, finding a feeder, and learning how to feed. Although this describes an extreme situation, it demonstrates the importance of adequate lighting from the first days of birds’ life, so that they can enjoy the opportunity to explore the environment, the other birds, and the resources available.

Inactive behaviour was not different between barn sides or across different age categories. According to some studies, this behaviour may be associated with increased bird age, walking ability deterioration, body weight, and fast growth rates [11,51,59]. Although our study did not test the birds’ walking ability, the number of culls regarding leg problems suggests that this problem was prevalent, causing suffering and pain to the birds, as well as limiting their behavioural repertoire.

Although light is an important element for birds, when provided in isolation, it may not be enough to reduce inactive behaviour. According to El-Deek and El-Sabrout [60], most of intensive production systems that are currently used do not usually support the natural behavioural needs of poultry. Therefore, farm animals may be reared in an environmental with enrichment and light which more closely resembles their natural characteristics. These options, acting together, may increase activity, improve leg health [11,61], and stimulate behaviours such as foraging and exploration [43]. However, selection for fast growth may lead to several welfare problems, such as metabolic disorders, decrease locomotor activity, and extend time spent sitting or lying [51]. For European Food Safety Authority (EFSA) [62], the risk assessment regarding poor welfare effects showed that fast growth is one of the major risk scores, including unbalanced body conformation, high stocking density, wet litter, and light intensity. Including slower-growing genetic strains may be a way to decrease welfare restrictions [51,63], adding to important environmental changes to indoor houses to meet the birds’ needs in the current poultry industry.

In general, animals engaged in pleasant activities, such as exploring, feeding, and interacting with other animals in a social group, may experience positive feelings, and without this engagement, the animal will not experience the full range of positive welfare states that are potentially available [64]. Although in our study, a qualitative behavioral assessment [30] was not used, it is likely that birds were more likely to experience positive feelings while they were in the NAL barn side, due to higher opportunities to increase behaviours that are more active, such as exploring, foraging, moving, and interacting with other birds.

Some behaviours were only also associated with age, such as comfort and feeding. Comfort behaviours were associated with increases in bird age categories. In the literature, this behaviour is associated to increases in chicken welfare, as the activities may related to the maintenance of bird health [65]. Alvino et al. [4] observed that broiler chickens reared in 5 lx spent less time in preening behaviour, as compared to those in 50 and 200 lx. The increase of comfort may be understood as positive results, indicating a possibility to encourage higher expression of behaviours associated to increases in chicken welfare. Although we only observed difference in feeding frequency regarding bird age categories, some authors observed a clear preference of laying hens and broiler chickens to eat in brighter lightings, from 20 to 200 lx [38,40], and that they ate more under 30 lx than 1 lx [66]. Birds may also find it aversive to eat in very dim light, because this behaviour is normally guided visually, and they see better in brighter environments [20,38]. Although feeding behaviour decreased with age in our study, no emaciated chicken was observed during the experimental period and feeding showed the second highest frequency, only behind inactive behaviour. It is also important to consider that, when the birds are foraging, they may also searching for feed [16], which may explain the lower number of visits to the feeder in the NAL side.

As for the “not visible” behavioural category, when the birds were younger, some of them stayed together in the passage between the barn sides, which may have given an enhanced sense of social interaction or protection. As birds aged, they may also have been looking for a different lighting, according to specific momentaneous needs. Birds observed in OAL spent less time in exploration, moving, and foraging, and when observed in this barn side, stayed lying very close or in front of each other, which also prevented appropriate behavioural identification. Thus, a potential reason for finding more birds in the not visible behaviour category in the OAL side may be an association between seeking an environment with lower light intensities and pen areas associated with a feeling of protection, provided by staying either close to wall angles in the passageways or close to another bird. Such potential reasons seem to indicate that the OAL side was chosen by the birds when they were searching for a cozy place to either rest or sleep.

## 5. Conclusions

For our experimental conditions, the chickens preferred natural and artificial lighting from 18 d of age onwards, when the confounding effect of the heating light was removed, and their behavioural repertoire was also different according to each side of the barn and to their ages. As the chickens also used the lower lit pen areas, barns with light gradient options seem important for them. In summary, the birds indicated that windows make a relevant difference in their indoor lives, as it is what they choose when the only other option is the same in-barn environment with only artificial lighting. Further preference studies are warranted to understand the potential effects of geographical, seasonal, climatic and genetic variations, amongst others.

## Figures and Tables

**Table 1:** Ethogram with definition of the behaviours recorded for broiler chickens during the preference test, performed from January to February 2021, in the State of Paraná, South of Brazil.

Behaviour	Definition
Feeding	Head in the feeder or pecking at the feed within the feeder
Drinking	Beak touching the drinker
Foraging	Pecking or scratching on the floor or both
Exploration or locomotion	Interacting with pen walls or locomotion behaviour, such as running, walking or jumping
Comfort	Preening, wing flapping, wing stretching, feather ruffling or shaking, and elements of dustbathing behaviour
Inactive	Sitting, lying or standing while not engaged in any activity, eyes open or closed
Not visible	Any behaviour that was not identified, due to birds standing very close or in front of each other or in the shielded part of passage ways between barn sides, resulting in an unsatisfactory recording angle

**Table 2:** Estimated preference probabilities and confidence intervals (CI) for Natural and Artificial Light (NAL) barn side according to bird age category, in a preference test performed from January to February 2021, in the State of Paraná, South of Brazil.

Bird Age Category		Preference		
Period	Observation Days	Estimates ^1^	CI (95%)	*p*-Value ^2^
I	9, 12, 15	0.538 ^a^	(0.435; 0.637)	0.470
II	18, 21, 24, 27	0.803 ^b^	(0.724; 0.864)	<0.001
III	30, 33, 36	0.627 ^a^	(0.523; 0.719)	0.016
	$\hat{\sigma }$^2^group = 0.169 (0.096); $\hat{\sigma }$^2^pen = 0.191 (0.090);			

^1^ Different letters mean different probabilities (*p* < 0.05); ^2^ *p*-value for testing null hypothesis that choice of barn side is random.

**Table 3:** Estimated probabilities for behaviours, according to the presence of windows (OAL vs. NAL) and broiler chicken age category (period I, II, and III), in a preference test performed from January to February 2021, in the State of Paraná, South of Brazil.

Behaviour	Effect				
	Bird Age Category		Window	Estimates ^3^	CI (95%)
	Period	Observation Days			
Feeding	I	9, 12, 15		0.343 ^a^	(0.279–0.413)
	II	18, 21, 24, 27	ns ^1^	0.275 ^ab^	(0.219–0.337)
	III	30, 33, 36		0.217 ^b^	(0.163–0.281)
$\hat{\sigma }$^2^group = 0.065 (0.091); $\hat{\sigma }$^2^pen = 0.073 (0.083); $\hat{\sigma }$^2^pen/day = 0.113 (0.070)					
Comfort	I	9, 12, 15		0.034 ^a^	(0.023–0.052)
	II	18, 21, 24, 27	ns ^1^	0.086 ^b^	(0.068–0.109)
	III	30, 33, 36		0.123 ^c^	(0.097–0.156)
$\hat{\sigma }$^2^group = 0.089 (0.077); $\hat{\sigma }$^2^pen = 0.367 (0.098); $\hat{\sigma }$^2^pen/day = 0.057 (0.067)					
Drinking	ns ^1^		OAL ^2^	0.026 ^a^	(0.016–0.041)
			NAL ^2^	0.045 ^b^	(0.035–0.059)
$\hat{\sigma }$^2^group = 0.208 (0.142); $\hat{\sigma }$^2^pen = 0.379 (0.146); $\hat{\sigma }$^2^pen/day = 0.535 (0.125)					
Exploration or locomotion	ns ^1^		OAL ^2^	0.031 ^a^	(0.020–0.049)
			NAL ^2^	0.053 ^b^	(0.042–0.068)
$\hat{\sigma }$^2^group = 0.493 (0.158); $\hat{\sigma }$^2^pen = 0.083 (0.099); $\hat{\sigma }$^2^pen/day = 0.191 (0.083)					
Not visible	ns ^1^		OAL ^2^	0.118 ^a^	(0.088–0.156)
			NAL ^2^	0.035 ^b^	(0.024–0.053)
$\hat{\sigma }$^2^group = 0.174 (0.125); $\hat{\sigma }$^2^pen = 0.327 (0.127); $\hat{\sigma }$^2^pen/day = 0.440 (0.110)					
Inactive	ns ^1^		ns ^1^	0.455	(0.416–0.494)
$\hat{\sigma }$^2^group = 0.053 (0.069); $\hat{\sigma }$^2^pen = 0.086 (0.059); $\hat{\sigma }$^2^pen/day = 0.122 (0.055)					

^1^ ns = not significant; ^2^ OAL = Only Artificial Light; NAL = Natural and Artificial Light; ^3^ Different letters mean different probabilities (*p* < 0.05).

**Table 4:** Estimated probabilities for foraging behaviour, according to the presence of windows (OAL vs. NAL) and broiler chicken age category, in a preference test performed from January to February 2021, in the State of Paraná, South of Brazil.

Bird Age Category		Window Presence	
Period	Observation Days	OAL ^1^	NAL ^1^
I	9, 12, 15	0.014 ^Aa^ (0.005; 0.037) ^2^	0.067 ^Ba^ (0.045; 0.097) ^2^
II	18, 21, 24, 27	0.009 ^Aa^ (0.002; 0.036) ^2^	0.007 ^Ab^ (0.004; 0.016) ^2^
III	30, 33, 36	0.019 ^Aa^ (0.006; 0.058) ^2^	0.009 ^Ab^ (0.003; 0.027) ^2^
	$\hat{\sigma }$^2^group = 0.343 (0.242); $\hat{\sigma }$^2^pen = 0.853 (0.271); $\hat{\sigma }$^2^pen/day = 0.453 (0.207)		

^1^ OAL = only artificial light; NAL = natural and artificial light; ^2^ Different capital letters refer to significant differences between barn sides (*p* < 0.05), and different low case letters indicate significant differences amongst birds’ age (*p* < 0.05).

## Data Availability

Data are available upon request from the corresponding author.
